# The sensori-motor model of the hippocampal place cells

**DOI:** 10.1186/1471-2202-16-S1-P2

**Published:** 2015-12-18

**Authors:** Anu Aggarwal

**Affiliations:** 1 Electrical and Computer Engineering Department, University of Maryland, College Park, MD, 20742, USA

## 

The hippocampal formation contains the head direction cells, the grid cells and the place cells which work as an internal GPS for the brain. The head directions cells can sense the direction in which the animal is moving, based on which the entorhinal grid cells fire at regular intervals as the animal is at the corners of an equilateral triangle and the hippocampal place cells [1] fire when the animal is at a place in the environment. Several mathematical models [2] have been proposed to explain firing pattern of the place cells, most of which consider place cell firing to be the result of integration of either sensory or processed motor inputs (received via the grid cells). Only the oscillatory interference model mentions the role of both sensory and motor inputs in place cell firing but does not explain how this information is remembered. However, empirical observations [2-6] indicate a role for both the sensory and the grid cell inputs in place cell firing and a one to one correspondence between the grid and the place cell firing patterns. Anatomical evidence [7] indicates that an area of the medial entorhinal cortex is primarily connected to a similar area of the hippocampus along the dorso-ventral axis. All the above is not possible if the place cell firing depended on integration of inputs from several grid cells or on integration of either sensory or motor inputs alone as proposed by the current models. Moreover, the animals without binocular vision, in whom most place cell recordings have been done, ought to have different mechanism for mapping the environment than those with binocular vision. Due to lack of depth perception, the former could be using the matrix formed by grid cells to hang the objects as seen by their eyes to get an accurate estimate of position. Therefore, we propose that the place cells integrate both sensory and motor inputs to them in a Bayes' optimal manner (Fig. 1). Also each place cell is connected to only one grid cell. As the animal enters an environment, the place cell resets. While the animal moves around in the environment, the grid cell firing causes movement along the ring of intermediate cells which wraps around. As soon as the sensory inputs identify the place to be of significance and cause a place cell to fire, by Hebbian learning, synapses develop between the place cell and the intermediate neuron which is firing concurrently in the ring. These synaptic connections help the animal to retrieve the location correctly during future encounters. This model offers improvements over the prior models as it explains the empirical observations more closely.

**Figure 1:**
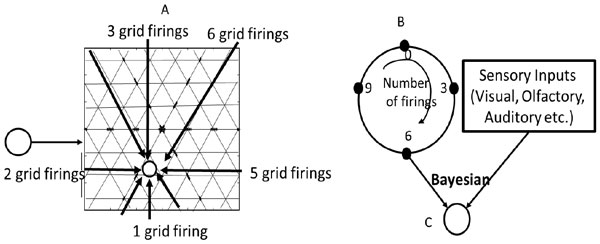
**Sensorimotor model of the hippocampal place cells**. A) Hexagonal grid cell firing gets integrated on to B) the ring of intermediate neurons. C) Together sensory and motor inputs are integrated in a Bayes' optimal manner on the place cell.

## References

[B1] O'Keefe J Conway DH Hippocampal Place Units in the freely moving rat: Why they fire where they fire Exp Brain Res 1978 31 573 590 658182 10.1007/BF00239813

[B2] Hasselmo ME How we remember 2012 1 MIT press

[B3] O'Keefe J Burgess N Dual phase and rate coding in hippocampal place cells: theoretical significance and relationship to entorhinal grid cells Hippocampus 2005 15 853 866 16145693 10.1002/hipo.20115 PMC2677681

[B4] Wills TJ Cacucci F Burgess N O'Keefe J Development of the Hippocampal Cognitive Map in Preweanling Rats Science 2010 328 1573 6 20558720 10.1126/science.1188224 PMC3543985

[B5] Chen G King JA Burgess N O'Keefe J How vision and movement combine in the hippocampal place code PNAS 2013 110 1 378 383 23256159 10.1073/pnas.1215834110 PMC3538268

[B6] Hafting T Fyhn M Bonnevie T Moser MB Moser EI Hippocampus independent phase precession in entorhinal grid cells Nature 2008 453 1248 1252 18480753 10.1038/nature06957

[B7] Witter MP Groenewegen HJ Lopes da Silva FH Lohman AHM Functional organisation of the extrinsic and intrinsic circuitry of the parahippocampal region Prog Neurobiol 1989 33 161 253 2682783 10.1016/0301-0082(89)90009-9

